# Global patterns in the metacommunity structuring of lake macrophytes: regional variations and driving factors

**DOI:** 10.1007/s00442-018-4294-0

**Published:** 2018-10-29

**Authors:** Janne Alahuhta, Marja Lindholm, Claudia P. Bove, Eglantine Chappuis, John Clayton, Mary de Winton, Tõnu Feldmann, Frauke Ecke, Esperança Gacia, Patrick Grillas, Mark V. Hoyer, Lucinda B. Johnson, Agnieszka Kolada, Sarian Kosten, Torben Lauridsen, Balázs A. Lukács, Marit Mjelde, Roger P. Mormul, Laila Rhazi, Mouhssine Rhazi, Laura Sass, Martin Søndergaard, Jun Xu, Jani Heino

**Affiliations:** 1 Geography Research Unit, University of Oulu, P.O. Box 3000, 90014 Oulu, Finland; 2 Finnish Environment Institute, Freshwater Centre, P.O. Box 413, 90014 Oulu, Finland; 3 Departamento de Botânica, Museu Nacional, Universidade Federal do Rio de Janeiro, Quinta da Boa Vista, Rio De Janeiro, RJ 20940‒040 Brazil; 4 Centre d’Estudis Avançats de Blanes (CEAB), Consejo Superior de Investigaciones Científicas (CSIC), C/accés a la Cala St. Francesc 14, 17300 Blanes, Spain; 5 National Institute of Water and Atmospheric Research Limited, P.O. Box 11115, Hamilton, New Zealand; 6 Centre for Limnology, Institute of Agricultural and Environmental Sciences, Estonian University of Life Sciences, 61117 Rannu, Tartumaa Estonia; 7 Department of Aquatic Sciences and Assessment, Swedish University of Agricultural Sciences (SLU), P.O. Box 7050, 750 07 Uppsala, Sweden; 8 Department of Wildlife, Fish and Environmental Studies, Swedish University of Agricultural Sciences (SLU), 901 83 Umeå, Sweden; 9 Tour du Valat, Research Institute for the conservation of Mediterranean wetlands, Le Sambuc, 13200 Arles, France; 10 Fisheries and Aquatic Sciences, School of Forest Resources and Conservation, Institute of Food and Agricultural Services, University of Florida, 7922 NW 71st Street, Gainesville, FL 32609 USA; 11 Natural Resources Research Institute, University of Minnesota Duluth, 5013 Miller Trunk Highway, Duluth, MN 55811 USA; 12 Department of Freshwater Protection, Institute of Environmental Protection‒National Research Institute, Krucza 5/11D, 00-548 Warsaw, Poland; 13 Department of Aquatic Ecology and Environmental Biology, Institute for Water and Wetland Research, Radboud University, Heyendaalseweg 135, 6525AJ Nijmegen, The Netherlands; 14 Department of Bioscience, Aarhus University, Vejsøvej 25, 8600 Silkeborg, Denmark; 15 Department of Tisza River Research, MTA Centre for Ecological Research, Bem tér 18/C, Debrecen, 4026 Hungary; 16 Norwegian Institute for Water Research (NIVA), Gaustadalléen 21, 0349 Oslo, Norway; 17 Department of Biology, Research Group in Limnology, Ichthyology and Aquaculture-Nupélia, State University of Maringá, Av. Colombo 5790, Bloco H90, CEP-87020-900 Mringá, PR Brazil; 18 Laboratory of Botany, Mycology and Environment, Faculty of Sciences, Mohammed V University in Rabat, 4 avenue Ibn Battouta, B.P. 1014 RP, Rabat, Morocco; 19 Faculty of Science and Technology, Department of Biology, Moulay Ismail University, PB 509, Boutalamine, Errachidia Morocco; 20 Illinois Natural History Survey, Prairie Research Institute, University of Illinois, 1816 South Oak Street, Champaign, IL 61820 USA; 21 Institute of Hydrobiology, Chinese Academy of Sciences, Wuhan, 430070 China; 22 Finnish Environment Institute, Biodiversity Centre, P.O. Box 413, 90014 Oulu, Finland

**Keywords:** Aquatic plants, Biogeography, Community structure, Elevation range, Environmental filtering, Hydrophytes, Metacommunity ecology, Spatial processes, Spatial variation

## Abstract

We studied community–environment relationships of lake macrophytes at two metacommunity scales using data from 16 regions across the world. More specifically, we examined (a) whether the lake macrophyte communities respond similar to key local environmental factors, major climate variables and lake spatial locations in each of the regions (i.e., within-region approach) and (b) how well can explained variability in the community–environment relationships across multiple lake macrophyte metacommunities be accounted for by elevation range, spatial extent, latitude, longitude, and age of the oldest lake within each metacommunity (i.e., across-region approach). In the within-region approach, we employed partial redundancy analyses together with variation partitioning to investigate the relative importance of local variables, climate variables, and spatial location on lake macrophytes among the study regions. In the across-region approach, we used adjusted *R*^2^ values of the variation partitioning to model the community–environment relationships across multiple metacommunities using linear regression and commonality analysis. We found that niche filtering related to local lake-level environmental conditions was the dominant force structuring macrophytes within metacommunities. However, our results also revealed that elevation range associated with climate (increasing temperature amplitude affecting macrophytes) and spatial location (likely due to dispersal limitation) was important for macrophytes based on the findings of the across-metacommunities analysis. These findings suggest that different determinants influence macrophyte metacommunities within different regions, thus showing context dependency. Moreover, our study emphasized that the use of a single metacommunity scale gives incomplete information on the environmental features explaining variation in macrophyte communities.

**Electronic supplementary material:**

The online version of this article (10.1007/s00442-018-4294-0) contains supplementary material, which is available to authorized users.

## Introduction

The continuing degradation of landscapes due to global change underscores the importance of understanding broad-scale patterns of biodiversity (Dudgeon et al. 2006; Vilmi et al. 2017). As a consequence, multi-discipline approaches are needed to understand biodiversity patterns and changes at various spatial scales. Biogeography and community ecology are two disciplines that share interests in investigating how historical events (e.g., glaciations), dispersal, biotic interactions, and environmental filtering structure biological communities at broad spatial and temporal extents (Brown and Lomolino 1998). Biogeography seeks to associate evolutionary, historical, and climatic influences on regional biota, and these biogeographic factors are typically strongly related to regional-scale diversity patterns (Svenning et al. 2008; Hortal et al. 2011). However, much uncertainty still exists in our understanding of the role of historical and climatic influences on local communities over broad extents, due in part to the lack of comparable data over large areas. Depending on the biological group and study region, the relative influence of history and climate vs. local environmental conditions on local community structure may differ. In some cases, history and climate have overcome the effects of local environmental conditions on local communities (Ricklefs and He 2016), whereas the opposite patterns have been found in other cases (Souffreau et al. 2015). Some studies have reported that both biogeographic characteristics and local environment have been important in explaining local community structure over broad spatial extents (Heino et al. 2017b; Rocha et al. 2017). These patterns can also be studied in the context of metacommunities, a discipline that connects biogeography and community ecology (Jenkins and Ricklefs 2011; Leibold and Chase 2018).

The main idea of metacommunity ecology is to understand the degree to which variation in local community structure is determined by environmental filtering and spatial dispersal processes (Winegardner et al. 2012; Heino et al. 2015b; Brown et al. 2016). The investigations of the relative contributions of these two processes are especially intriguing in lakes, which are island-like systems surrounded by terrestrial land uninhabitable for aquatic organisms (Hortal et al. 2014). Therefore, dispersal is challenging for species relying on watercourse connections for movement among lake habitats, although humans have acted as dispersal vectors for many organisms (see, e.g., Heino et al. 2017a). A recent meta-analysis also suggested that the importance of environmental filtering is the lowest in lakes when compared to other terrestrial and more connected aquatic ecosystem types (Soininen 2014). Other lake studies have found that biological assemblages with passive dispersal mode or large body size are more structured by spatial processes than local environmental conditions (Beisner et al. 2006; De Bie et al. 2012; Padial et al. 2014). However, a large amount of variation is present in the findings depending on the studied biological group, study region, and spatial extent, leading to context dependency in the patterns detected (Alahuhta and Heino 2013; Tonkin et al. 2016). One biological group showing context dependency has been aquatic macrophytes, many of which are distributed around the world due to efficient dispersal abilities and colonization strategies (Santamaría 2002; Chambers et al. 2008). Environmental filtering has thus often overruled spatial factors in explaining variation in macrophyte community structure (Capers et al. 2010; Mikulyuk et al. 2011; Alahuhta et al. 2013; Viana et al. 2014), although opposite patterns have been found in some metacommunities (Hájek et al. 2011; Padial et al. 2014). These conflicting patterns for aquatic macrophyte metacommunities call for a more holistic comparative analysis including data sets with identical explanatory variables from different regions globally.

Aquatic macrophytes often show large-scale biodiversity patterns that deviate from those found in many other biological groups. For example, although the latitudinal diversity gradient (i.e., the decrease in the number of species from the Equator to the poles) has been found for numerous biological groups in different ecosystems (Kinlock et al. 2018), macrophyte diversity often peaks at intermediate latitudes (Chappuis et al. 2012; Crow 1993). At regional extents, macrophyte diversity may show conflicting patterns in relation with latitude depending on the study region. For example, macrophytes have followed the latitudinal gradient in the Fennoscandia (Alahuhta et al. 2013), whereas a reversed pattern has been evidenced in the Midwestern USA (Johnston et al. 2010; Alahuhta 2015). Aquatic macrophytes may respond to climatic and elevational gradients at broad spatial scales, but these broad-scale characteristics are typically overcome by local environmental factors when accounting for variation in community structure (Kosten et al. 2011; Alahuhta 2015). For example, the macrophyte diversity–lake area relationship has varied from strongly positive to non-significant among studies conducted thus far (Jones et al. 2003; Hinden et al. 2005), likely because lake area may poorly describe the diversity–area relationship in deep lakes, where a large proportion of the lake is uninhabitable for macrophytes (Søndergaard et al. 2013). Depth gradient has often been negatively associated with macrophyte diversity, because the availability of light in water dictates photosynthesis rate for aquatic macrophytes (Kosten et al. 2009b; Søndergaard et al. 2013). Macrophytes also typically respond strongly to lake water chemistry (e.g., Chappuis et al. 2014). For example, aquatic macrophyte diversity has shown linear or unimodal in relation with total phosphorus, possibly because it is the primary nutrient for freshwater primary producers (Elser et al. 2007; Kosten et al. 2009a). However, it is difficult to draw comprehensive conclusions regarding how these environmental gradients structure aquatic macrophyte communities, due to inconsistencies among the studies (e.g., differences in spatial scales, explanatory variables, and methods used). Thus, investigations executed with identical study designs across multiple study sites and regions are needed to enhance our understanding of the relationships between aquatic macrophytes and environmental gradients (e.g., Borer et al. 2014; Heino et al. 2015a; Alahuhta et al. 2017a).

**Fig. 1:**
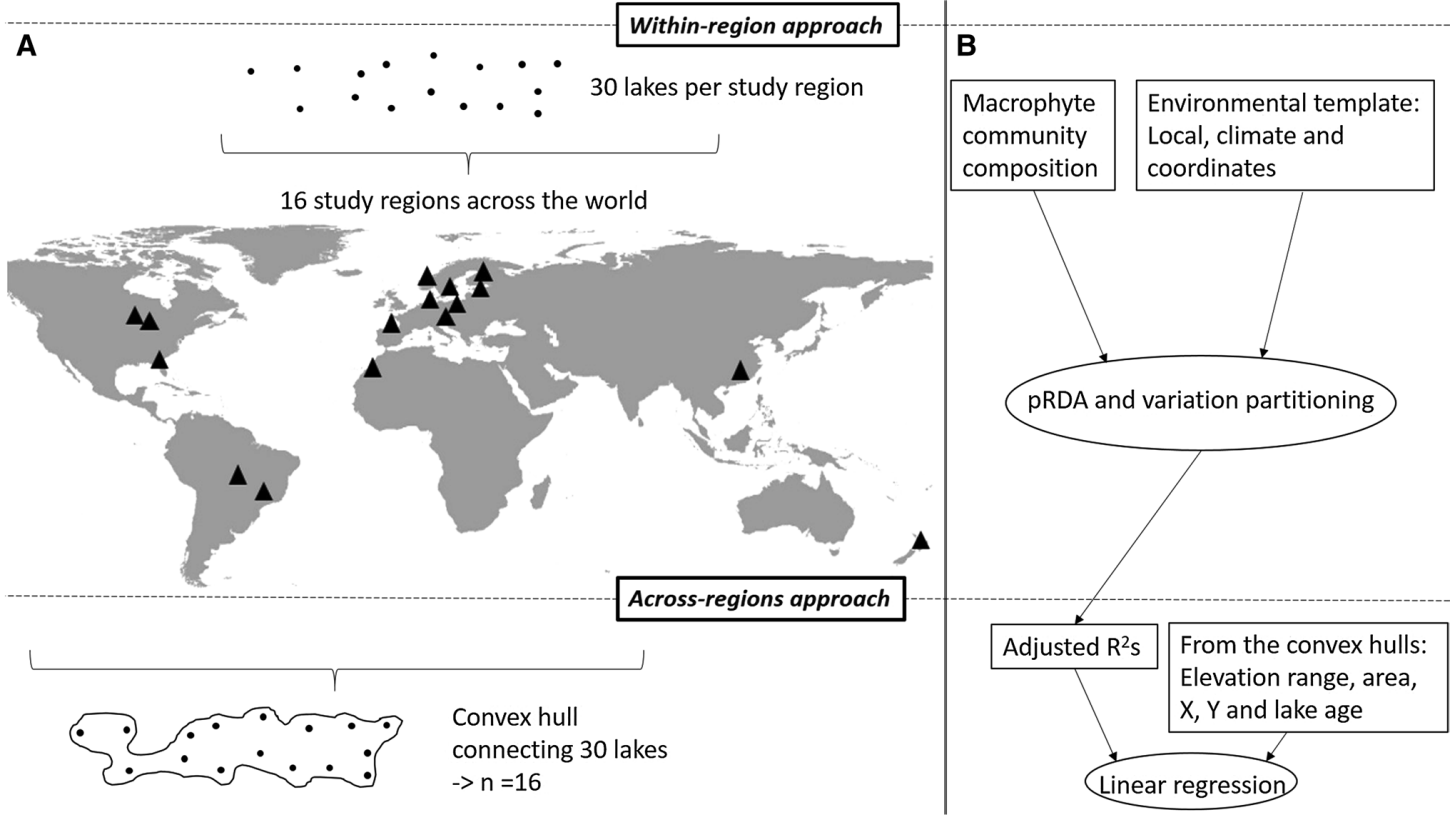
Our study system comprised ca. 30 lakes surveyed in 16 metacommunities (black triangles) across the world. In the regional study approach, a convex hull that connected all 30 lakes in a region was drawn for each metacommunity separately, enabling us to obtain explanatory variables from the convex hull (**a**). We investigated lake macrophyte communities in relation with local variables, climate variables and lake coordinates separately in each metacommunity using partial redundancy analysis (pRDA) and variation partitioning (VP). Adjusted *R*^2^ values gained from the VP for pure local and climate variables in addition to lake coordinates and full model including all three environmental variable groups were used as response variables in the across-region approach (*N* = 16). The adjusted *R*^2^ values were regressed against a set of environmental variables (i.e., elevation range, area, geographic coordinates and estimated maximum lake age), which were obtained from a convex hull for each metacommunity (**b**). Metacommunity refers to ‘within-region approach’ and regional to ‘across-region approach’

**Fig. 2:**
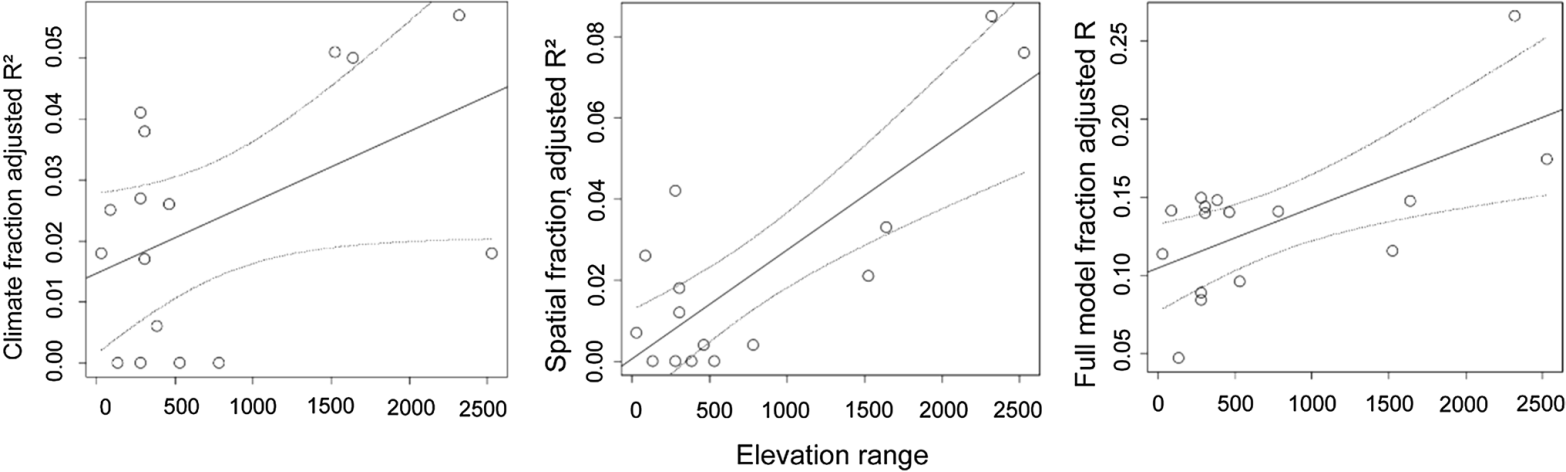
Relationships between the adjusted *R*^2^ values obtained through variation partitioning of pure climate fraction, spatial location fraction and full model of freshwater macrophytes and elevation range (*N* = 16)

The overall purpose of this study was to investigate the community–environment relationships of lake macrophytes at the metacommunity scale using data sets collected from all over the world. More specifically, we studied (1) whether the lake macrophyte communities respond similar to key local environmental factors, major climate variables, and lake spatial locations in 16 study regions covering six continents (i.e., within-region approach, Figs. 1, 2) how well can explained variability in the community–environment relationships across multiple lake macrophyte metacommunities be accounted for by elevation range, spatial extent, latitude, longitude, and age of the oldest lake within each metacommunity (i.e., across-region approach, Fig. 1). Based on the previous findings on lake macrophyte metacommunities from different regions (e.g., Capers et al. 2010; Mikulyuk et al. 2011; Alahuhta et al. 2013), we expected that environmental filtering should dominate over spatial factors in explaining macrophyte community structure, and this would be more apparent in stable and old lakes (i.e., of glacial origin) than in unstable young lakes, such as floodplain lakes. Because elevation range contributed strongly to global macrophyte turnover in a recent study (Alahuhta et al. 2017a), we hypothesized that elevation range would explain a large amount of variation in the across-region approach including multiple macrophyte metacommunities. Following the findings from a recent meta-analysis that a latitudinal diversity gradient does not exist for freshwater assemblages (Kinlock et al. 2018), we did not expect to find a significant relationship between the strength of community–environment relationships of macrophytes and latitude in the across-region approach. Finally, many terrestrial plants and trees have been shown to respond to historical effects, including the last glacial maximum (Svenning et al. 2008; Ordonez and Svenning 2016), and some studies have suggested that historical effects may be important also for macrophytes as well (Alahuhta et al. 2018). Based on this combined evidence, we suggest that the historical effect may have some influence on the strength of the community–environment relationships in the across-region approach.

## Materials and methods

### Macrophyte data

We surveyed lake macrophytes in 16 different regions covering six continents across the Earth (Table 1). Overall, 27–30 lakes were investigated in each region. In each region, we randomly chose ca. 30 lakes with similar geographical distribution from the pool of candidate lakes. The selected lakes ranged from floodplain lakes in Brazil and China to glacial-origin relatively stable lakes situated at boreal and temperate zones (e.g., Finland, Estonia, Sweden, Norway, Denmark, New Zealand, Poland, and US states of Minnesota and Wisconsin). Although the lakes differed in their environmental conditions among the regions, all lakes were mostly natural lentic systems (i.e., reservoirs were excluded). However, most of the lakes suffered from various anthropogenic pressures such as nutrient enrichment, alien invasive species, water-level fluctuations, and decreased connectivity. The inclusion of different types of lakes was considered an important factor increasing the range of environmental conditions, resulting in environmental filtering effects. Detailed descriptions of study lakes can be found in the Supporting Information (Appendix S1).

**Table 1:** Descriptive statistics of the studied lakes and convex hulls and their environmental conditions

Study region	Number of lakes	Number of taxa	Species level identification of all taxa (%)	Local variables in the with-region approach			Climate variables in the within-region approach			Explanatory variables in the across-region approach				
				Total phosphorus (mg/l)	Secchi depth (m)	Lake area (km^2^)	Temperature, mean (°C)	Temperature range (°C)	Precipitation (mm)	Elevation range of convex hull (m)	Area of convex hull (km^2^)	Latitude of convex hull	Longitude of convex hull	Estimated maximum lake age within convex hull/order
Brazil, Parana river floodplain	28	37	89.2	0.04 (0.02–0.12, 0.02)	1.14 (0.60–2.09, 0.38)	17.43 (0.01–113.8, 30.97)	22.89 (22.80–23.00, 0.06)	19.69 (19.50–20.00, 0.14)	1308.36 (1270.00–1378, 37.47)	33.00	262.10	− 22.78	− 53.39	4000/10
Brazil, coastal lakes	28	18	94.5	0.08 (0.02–0.30, 0.06)	1.44 (0.15–4.30, 1.21)	46.16 (10.66–127.23, 30.82)	24.80 (22.90–27.20, 1.21)	13.26 (11.20–16.40, 1.25)	1102.86 (590.00–1382.00, 183.94)	1525.00	39762.60	− 13.26	− 38.98	1000/11
China	30	32	100	0.13 (0.02–0.45, 0.12)	1.75 (0.17–9.23, 2.79)	175.56 (10.20–2933.00, 688.40)	17.01 (15.40–18.20, 0.56)	32.43 (30.90–33.10, 0.65)	1288.30 (1012.00–1577.00, 128.00)	523.00	22243.70	30.12	115.13	35,000,000/1
Denmark	30	50	98	0.12 (0.01–0.71, 0.15)	1.66 (0.02–3.81, 0.98)	2.20 (0.01–39.54, 7.42)	7.39 (7.00–7.70, 0.20)	24.46 (21.50–25.6, 1.02)	734.27 (587.00–872.00, 60.03)	137.00	2961.80	56.17	9.58	22,000/4
Estonia	28	49	100	0.04 (0.01–0.13, 0.03)	1.83 (0.4–4.2, 0.99)	191.72 (8.00–943.6, 193.25)	5.07 (4.40–5.80, 0.36)	30.70 (28.10–32.10, 1.01)	631.21 (588.00–693.00, 24.50)	283.00	33549.30	58.57	25.50	10,000/7
Finland	29	52	100	0.03 (0.01–0.18, 0.04)	2.14 (0.02–5.00, 1.42)	24.99 (0.25–328.98, 71.59)	2.76 (0.60–4.40, 0.93)	34.18 (30.90–36.60, 1.32)	606.69 (535.00–669.00, 34.64)	308.00	101066.10	62.46	26.37	9500/8
Florida (US state)	28	36	100	0.03 (0.01–0.16, 0.03)	2.02 (0.39–6.65, 1.62)	11.26 (0.04–121.88, 23.78)	21.34 (19.30–22.50, 1.01)	25.10 (21.10–28.90, 2.04)	1296.57 (1202.00–1563.00, 83.11)	91.00	32675.10	28.72	− 82.07	10,000/7
Hungary	30	36	100	0.11 (< 0.01–0.46, 0.09)	0.80 (0.20–2.90, 0.55)	1.10 (0.02–10.34, 1.87)	10.58 (9.80–11.30, 0.42)	31.89 (30.60–32.70, 0.62)	554.97 (517.00–652.00, 39.09)	283.00	14573.20	47.10	20.38	500/12
Minnesota (US state)	30	51	88	0.03 (0.01–0.09, 0.02)	2.65 (0.60–7.90, 1.54)	4.12 (0.05–30.89, 6.24)	3.67 (2.60–5.20, 0.79)	46.49 (41.90–48.80, 2.05)	718.63 (673.00–770.00, 26.94)	465.00	41787.90	47.14	− 92.80	20,000/5
Morocco	29	43	100	0.28 (0.05–0.84, 0.18)	0.43 (0.09–1.15, 0.26)	0.60 (< 0.01–4.00, 1.07)	14.80 (9.20–19.10, 3.11)	28.75 (20.70–35.80, 5.23)	674.41 (451.00–1087.00, 206.85)	2322.00	8557.90	34.29	− 5.69	25,000/3
New Zealand	30	39	100	0.02 (0.01–0.06, 0.01)	5.16 (1.66–16.03, 3.12)	3254.12 (2.06–61264.45, 11367.65)	13.79 (9.30–16.30, 2.06)	18.14 (15.70–22.70, 2.06)	1433.80 (806.00–3858.00, 516.08)	1640.00	19824.00	− 37.80	175.60	50,000/2
Norway	29	26	92.4	0.03 (< 0.01–0.20, 0.04)	2.79 (0.50–6.90, 1.45)	0.11 (0.01–0.58, 0.11)	5.23 (3.80–5.50, 0.34)	21.32 (20.58–21.10, 0.44)	1571.10 (1364.00–1765.00, 81.09)	309.00	347.00	64.91	11.36	10,000/7
Poland	28	43	100	0.07 (0.01–0.38, 0.08)	2.14 (0.40–4.90, 1.68)	2.41 (0.58–6.61, 1.63)	7.52 (5.90–9.00, 0.81)	29.81 (26.00–32.00, 1.21)	589.50 (524.00–681.00, 53.57)	284.00	54227.60	53.24	18.13	22,000/4
Spain	30	10	100	<0.01 (< 0.01–0.02, < 0.01)	4.69 (1.00–12.00, 3.03)	3.24 (0.30–14.40, 3.50)	2.28 (0.50–3.60, 0.82)	22.35 (22.10–22.70, 0.21)	1370.47 (1290.00–1473.00, 50.12)	2531.00	4733.20	42.68	0.84	10,000/7
Sweden	29	56	100	0.02 (< 0.01–0.06, 0.02)	2.66 (0.67–5.90, 1.32)	2.03 (0.02–15.81, 3.07)	5.05 (1.00–7.90, 2.09)	28.93 (22.90–37.60, 3.79)	613.14 (514.00–853.00, 90.14)	784.00	137543.70	60.83	15.97	9000/9
Wisconsin (US state)	29	63	95.3	0.03 (0.01–0.14, 0.03)	2.52 (0.91–5.00, 0.97)	0.56 (0.19–1.36, 0.33)	5.98 (3.90–8.20, 1.82)	42.75 (38.70–47.60, 2.05)	814.28 (707.00–878.00, 34.90)	387.00	26671.30	44.11	− 89.06	19,000/6

For the columns from total phosphorus to precipitation, the values are mean (min–max, SD). The actual estimated maximum lake ages (left side value) were changed to a ranked variable ranging from the youngest to oldest (right side value), because there was no information on the maximum age estimates for all 30 lakes in each region and the age estimates showed high level of variation in some study regions

The macrophyte data consisted of presence–absence observations of hydrophyte species, i.e., species which grow exclusively in freshwaters. These hydrophytes consisted of submerged (elodeids and isoetids), free-floating (ceratophyllids and lemnids), floating-leaved, and emergent species (Cook 1999). Emergent hydrophytes included only those species strongly bound to aquatic environments and found to grow in water at the time of survey, like *Alisma plantago*-*aquatica*, *Butomus umbellatus*, *Glyceria fluitans*, *Juncus bulbosus*, *Mentha aquatica*, *Sagittaria sagittifolia,* and *Schoenoplectus lacustris* (Tanner et al. 1986; Crow 1993; Willby et al. 2000; Thomaz et al. 2003; Kosten et al. 2009a). In addition to non-aquatic emergent and shore species, charophytes and aquatic bryophytes were removed from the data sets, because only hydrophytes were exclusively surveyed in all the regions. We also excluded hybrids, sub-species, and genus level identifications when species from the same genus were recorded from the data. We refer to this set of aquatic species as macrophytes hereafter. All macrophytes were empirically surveyed using similar methods within each region. This enabled us to compare the strength of the community–environment relationships across the 16 regions and to minimize the potential negative influences caused by different survey methods within each region. The macrophyte surveys were executed mostly between 2001 and 2013. The exceptions were Norway and US states of Florida and Minnesota, which were surveyed in 1998, between 1991 and 2013, and between 1992 and 2003, respectively.

### Explanatory data: within-region approach

To explore which factors explain the variability in macrophyte community structure within a region (a single metacommunity), we compiled three groups of lake-level variables: local variables, climate variables, and spatial location (Table 1). Local variables consisted of water total phosphorus concentration (mg/l), Secchi depth (m), and lake area (km^2^). Secchi depth indicates various ecological responses, ranging from eutrophication to amount of humic substances in water and visibility (Chambers and Kalf 1984; Kosten et al. 2009b). Lake area is typically used to mirror species–area relationship for aquatic organisms (Jones et al. 2003; Alahuhta et al. 2013), but lake area does not necessarily comprehensively indicate this relationship in lakes, where a large extent of the lake is too deep for macrophyte colonization and growth (Mikulyuk et al. 2011; Søndergaard et al. 2013). However, data on maximum colonization depth were not available for all study lakes. Moreover, lake area is often highly correlated with shoreline length which mirrors species–area relationship relatively well for many aquatic organisms (Søndergaard et al. 2005; Lewin et al. 2014). These three local variables are among the most important explaining variation in lake macrophyte community structure, and often correlate with other water chemistry and hydromorphological variables that were not available for all the study lakes (Jones et al. 2003; Lacoul and Freedman 2006; Kosten et al. 2009a). Local variables were surveyed and determined similarly within each study region (Appendix S1).

Climate variables comprised atmospheric annual mean temperature (°C), annual temperature range (°C), and annual precipitation defined for each study lake based on 30 year average values obtained from the WorldClim (Hijmans et al. 2005). Annual mean air temperature was used as a proxy for thermal energy availability for macrophytes, whereas annual temperature range represented variation in thermal energy availability and its annual distribution in study lakes in different parts of the world (Kosten et al. 2009a; Alahuhta et al. 2017a). Annual precipitation was not only a surrogate for water-level fluctuation (incl. flooding and drying events) and potential dispersal via watercourses, but also for nutrient and material loading from the catchment (Soons et al. 2008; Carpenter et al. 2011). Climate variables were determined for each lake’s center coordinate from 1 km resolution data, because it was not possible to extract values for a whole lake due to small surface area (i.e., < 1 km^2^) in many of the studied water bodies. Although we used atmospheric temperatures, they follow closely surface water temperatures across the world (O’Reilly et al. 2015).

Different methods, ranging from simple coordinates and trend surface analysis to principal coordinates of neighbor matrices analysis (PCNM), have been used to quantify spatial processes such as dispersal limitation (see a review for the freshwater realm, Heino et al. 2017a). However, none of these methods has proven superior for distinguishing spatial processes for local communities, especially when combined with variance partitioning (Gilbert and Bennett 2010; Smith and Lundholm 2010). In our work, geographic coordinates of lake centers were used to represent spatial locations among the 30 selected lakes within each study region; therefore, we utilized geographic coordinates, because we were interested only in broad-scale spatial patterns among the lakes. More importantly, we wanted to balance the study design by including the same number of environmental variables in each of the three lake-level explanatory variable groups to avoid type I error (Burnham and Anderson 2002). For example, the use of principal coordinates of neighbor matrices (PCNMs) analysis would have resulted to variable number of spatial variables in each study region, flawing our study design (e.g., Gilbert and Bennett 2010). However, to compare the results of these two methods (geographic coordinates vs. PCNMs) to obtain spatial variables, we also calculated PCNMs based on Euclidean distances among lakes separately in each metacommunity (Borcard and Legendre 2002).

### Explanatory data: across-region approach

To investigate which characteristics structure the variability in macrophyte community structure across all regions (multiple metacommunities), we summarized regional environmental information within convex hulls encompassing the minimum area containing all surveyed lakes within each of the 16 regions (Heino et al. 2015a; Alahuhta et al. 2017a). For each study region, we defined elevation range within the convex hull (m), area of the convex hull (km^2^), latitude of the convex hull (from centroid), longitude of the convex hull (from centroid), and estimated the maximum age of the oldest lake within a particular study region (Table 1). Elevation range represented variability in habitats suitable for macrophytes and indicated temperature variation within a region (Wang et al. 2011; Alahuhta et al. 2017a). Elevation range was not sensitive to extreme values, as elevation range and quantile elevation range were significantly correlated (*R*_Spearman_: 0.75, *p* = 0.0009). The convex hull area was used as a proxy for environmental heterogeneity (Gaston 2000). Both latitudinal and longitudinal gradients are known to affect freshwater species distributions (Chappuis et al. 2012; Griffiths et al. 2014). Longitude can indirectly affect macrophytes by indicating variation in large-scale climate (e.g., marine vs. continental climate), natural geological, soil or habitat properties, and land use changes (Kosten et al. 2009a; Sass et al. 2010; Alahuhta et al. 2017b). The age of the oldest lake was used as a surrogate for temporal availability of colonization sources for macrophyte species within each region. These estimates were based on literature and/or sediment dating. However, there was no information on the maximum age estimates for all 30 lakes in each region and there was high variation in the age estimates in some study regions (e.g., based on sediment dating). For this reason, we considered that (a) it would not be possible to use lake-specific age estimates in the within-region approach, and (b) high variation in the actual values of age estimates would lead to serious lack of precision in the across-region approach. To overcome this problem, we changed the actual age estimations to a ranked variable ranging from the youngest (one) to oldest (12). Quadratic terms of these explanatory variables on the macrophytes were tested in the analysis, but these were not significant and were thus excluded from the analysis.

### Statistical analysis

In the within-region (a single metacommunity) approach, we utilized partial redundancy analyses (pRDA) to distinguish the relationships between variation in macrophyte community composition and the three explanatory variable groups (i.e., local variables, climate variables, and spatial location), following the well-established variation partitioning protocol (Borcard et al. 1992). The species matrices were Hellinger-transformed prior to the RDAs to increase linearity of the studied gradients (Legendre and Gallagher 2001). Total variation in macrophyte community composition was partitioned into three independent and four shared fractions: (1) pure local variables; (2) pure climate variables; (3) pure spatial location; (4–7) their shared fractions; and (8) unexplained. The detailed procedures needed to calculate these fractions have been explained previously in the literature (Anderson and Cribble 1998; Borcard et al. 2011). As our main study purpose was to assess the relative importance of local variables, climate variables and spatial location among the study regions, we conducted variation partitioning separately for the 16 study regions using the same environmental variables. All environmental variables were forced in the pRDAs to maintain comparability among the study regions and to gain equal amount of information for the regional study approach (see below). The variation explained by each of the three variable group was evaluated using adjusted R^2^, which gives unbiased estimates of the explained variation (Peres-Neto et al. 2006). In addition, variation partitioning based on pRDA following the protocol described above was separately conducted between macrophyte community composition and local variables, climate variables, and PCNMs to find out whether the influence of spatial location differed when using either geographic coordinates or PCNMs. The suitable number of positively autocorrelated PCNMs was selected using the protocol of Blanchet et al. (2008), where all local and climate variables were forced in the models. The variation partitioning results (based on PCNMs) were not utilized in the across-region approach for the reasons explained above (in explanatory data: within-region approach). The pRDAs and variation partitioning procedures were performed in the R environment with the vegan (Oksanen et al. 2013) and packfor (Blanchet et al. 2008) packages.

In the across-region (multiple metacommunities) approach, we used adjusted R^2^ values obtained from the pure fractions of variation partitioning (separately for the pure local, climate, and spatial variables, and for a full model including all variables) for each of the 16 study regions as response variables to study how the strength of the macrophyte community–environment relationships vary across the study regions. We used simple linear regression between the adjusted *R*^2^ values and all environmental gradients (i.e., elevation range, area, latitude, longitude, and estimated maximum lake age within convex hulls) in the further analysis. Adjusted *R*^2^ values of pure local variables were arcsine square root transformed prior to the analysis to achieve normality. To get additional information on the order of importance of different environmental gradients on the macrophytes across the study regions, we utilized commonality analysis to decompose linear regression effects to unique and common components (Nathans et al. 2012). The unique effects suggest how much variance is solely explained by a single explanatory variable, whereas common effects indicate how much variance is shared by two or more explanatory variables together (Ray-Mukherjee et al. 2014). A higher value of common effects compared to unique effect also suggests a greater collinearity among explanatory variables (Nathans et al. 2012; Ray-Mukherjee et al. 2014). In addition, negative values can occur in the common effects if some of the relationships among environmental variables have opposite trends (Ray-Mukherjee et al. 2014). Compared to other similar statistical methods, commonality analysis is independent of variable order that can disturb, for example, stepwise multiple regression results (Nathans et al. 2012; Petrocelli et al. 2003). Besides unique and common effects, we produced beta and structure coefficients. Beta coefficients indicate an environmental variable’s total contribution to the regression equation, whereas structure coefficients are bivariate correlations between a predictor variable and the dependent variable’s score resulting from the regression model (Nathans et al. 2012; Ray-Mukherjee et al. 2014). Unlike beta coefficients, structure coefficients are independent of collinearity among predictor variables (Ray-Mukherjee et al. 2014). Commonality analysis was executed using the ‘yhat’ package (Nimon et al. 2013) in the R environment.

## Results

### Within-region approach

The overall explained variation varied from 4.7% in Denmark to 26.6% in Morocco (Table 2). Of the pure fractions, local variables were most important for macrophyte metacommunities in 9 out of 16 regions. The explained variations of these pure local environmental fractions differed from 0.9% in Poland to 10.5% in China. The highest effect of pure fractions of climate variables was on metacommunities in Brazil coastal lakes (5.1%) and New Zealand (5.1%), while the highest effect of spatial location was on metacommunities in Morocco (8.5%) and Spain (7.6%). In addition, pure fractions of local and climate variables were equally high in the US states of Minnesota (2.4% and 2.6%, respectively) and Wisconsin (0.90% and 0.56%, respectively), whereas pure effects of climate (4.1%) and spatial location (4.1%) contributed similarly in Estonia. In addition, many joint fractions showed high-explained variation for macrophytes.

**Table 2:** Results of the variation partitioning (results shown as adjusted *R*^2^ values × 100) based on partial redundancy analysis (pRDA) in explaining the relationship between lake macrophyte communities and three environmental variable groups (i.e., local variables, climate variables and geographical variables) in each study region

	Local variables (LV)	Climate variables (CV)	Spatial location (XY)	LV + CV	CV + XY	LV + XY	LV + CV + XY	Unexplained variation
Brazil, Parana river floodplain	2.07	1.83	0.68	− 1.81	4.12	− 0.81	5.27	88.65
Brazil, coastal lakes	1.71	5.05	2.09	3.35	− 1.69	− 0.66	1.70	88.45
China	**10.46**	0.00	0.00	− 2.56	4.43	0.25	0.33	90.41
Denmark	3.87	0.00	0.00	0.22	1.77	1.42	− 0.51	95.28
Estonia	0.89	**4.12**	**4.18**	3.23	1.08	3.59	− 2.11	85.02
Finland	**5.23**	1.65	1.78	3.25	1.27	0.04	0.79	86.00
Florida	**9.94**	2.49	2.56	3.10	− 3.21	2.22	− 2.95	85.85
Hungary	3.21	2.67	0.00	− 1.02	5.75	0.60	− 0.04	91.10
Minnesota	2.43	2.63	0.36	0.19	6.94	− 0.21	1.69	85.97
Morocco	2.54	**5.66**	**8.53**	0.93	5.79	0.04	3.11	73.40
New Zealand	1.66	**5.05**	3.31	− 0.76	3.38	− 0.89	3.01	85.23
Norway	**7.76**	3.79	1.16	− 1.44	2.14	− 2.69	3.66	85.62
Poland	1.04	0.00	− 1.95	2.96	5.42	1.56	0.49	91.60
Spain	**6.06**	1.81	**7.55**	1.17	1.80	− 1.82	0.85	82.58
Sweden	**7.17**	0.00	0.37	3.40	5.36	0.28	− 1.20	85.91
Wisconsin	0.90	0.56	0.00	− 0.24	12.94	0.52	0.23	85.21

Separate pRDA analysis using identical explanatory variables was done for each study region. Significant (*p* < 0.05) pure fractions are bolded

The joint effect of climate and spatial location was very important for macrophyte metacommunities in Brazil’s Parana river floodplain (4.1%), Hungary (5.8%), Minnesota (6.9%), Poland (5.4%), and Wisconsin (12.9%). Joint influence of all the three variable groups in Brazil’s Parana river floodplain (5.3%) and local and climate variables in Poland (3.0%) explained considerable amount of variation for macrophytes. Other joint effects also showed a great amount of variation in China, Estonia, Morocco, New Zealand, and Sweden, but they were not as important as pure fractions. Different individual variables were significant for macrophyte metacommunities in different study regions (Appendix S2).

Variation partitioning results using PCNMs as indicators of spatial influences differed to some extent from corresponding analyses, where spatial location was based on geographic location (Appendix S3). The contribution of pure spatial location based on PCNMs was higher than that based on geographic coordinates in China, Finland, Florida, Hungary, Morocco, and Norway. The opposite pattern was found in Estonia, Salga project lakes, and Spain. However, all selected PCNMs were first eigenvectors (Appendix S4), which indicate broad-scale variation in spatial patterns similar to that of geographic coordinates. We do not debate these results further due to potential issues elaborated in “Materials and methods”.

### Across-region approach

The linear regression models (regional variables vs. the explained variance in macrophyte community composition in the within-region variation partitioning) modestly explained the overall variation in macrophyte community composition in the across-region approach (Table 3). The adjusted *R*^2^ from the linear models ranged from 0 (multiple *R*^2^ 0.10) for the pure local fraction of the variation partitioning to 0.56 (multiple *R*^2^ = 0.70) for the pure spatial location of the variation partitioning. These low overall explained variations were to be expected due to the small number of regions (*n* = 16); however, we were most interested in whether, and to what extent, the regional explanatory variables would contribute to macrophytes in the across-region approach. None of the predictor variables significantly explained the pure local fraction.

**Table 3:** Results of commonality analysis for each environmental variable based on regression models for pure local adjusted *R*^2^ values, pure climate adjusted *R*^2^ values, pure broad-scale spatial pattern adjusted *R*^2^ values, and full model adjusted *R*^2^ values

Environmental variable	Estimate	SE	*t*	*p*	*β*	SC	Unique	Common	Total
Pure local adj. *R*^2^									
Elevation range	< 0.001	< 0.001	− 0.015	0.988	− 0.005	− 0.165	< 0.001	0.003	0.003
Area	< 0.001	< 0.001	0.103	0.920	0.034	0.410	0.001	*0.016*	0.017
*X*	< 0.001	< 0.001	0.559	0.588	0.198	0.402	0.028	− 0.012	0.016
*Y*	0.001	0.001	0.838	0.422	0.284	0.830	**0.063**	0.002	0.065
Lake age	0.001	0.009	0.065	0.949	0.023	− 0.169	< 0.001	0.003	0.003
Pure climate adj. *R*^2^									
Elevation range	< 0.001	< 0.001	1.567	0.148	0.416	0.768	**0.150**	0.079	0.229
Area	< 0.001	< 0.001	− 0.890	0.394	− 0.243	− 0.506	0.049	0.051	0.100
*X*	< 0.001	< 0.001	0.190	0.853	0.055	0.130	0.002	0.004	0.007
*Y*	< 0.001	< 0.001	− 0.740	0.476	− 0.207	− 0.664	0.034	*0.138*	0.171
Lake age	0.002	0.002	− 0.841	0.420	0.245	0.146	0.043	− 0.035	0.008
Pure spatial location adj. *R*^2^									
Elevation range	< 0.001	< 0.001	4.613	0.001**	0.851	0.961	**0.630**	0.020	0.650
Area	< 0.001	< 0.001	− 0.952	0.363	− 0.180	− 0.292	0.027	*0.033*	0.060
*X*	< 0.001	< 0.001	− 0.351	0.733	− 0.071	0.095	0.004	0.003	0.006
*Y*	< 0.001	< 0.001	0.938	0.370	0.183	− 0.130	0.026	− 0.014	0.012
Lake age	< 0.001	0.002	0.032	0.975	0.006	− 0.185	0.000	*0.024*	0.024
Full model adj. *R*^2^									
Elevation range	< 0.001	< 0.001	3.124	0.011**	0.729	0.890	**0.462**	− 0.044	0.418
Area	< 0.001	< 0.001	0.429	0.677	0.103	− 0.022	0.009	− 0.009	< 0.001
*X*	< 0.001	< 0.001	− 1.295	0.225	− 0.332	− 0.141	0.079	− 0.069	0.011
*Y*	< 0.001	< 0.001	0.354	0.731	0.087	− 0.040	0.006	− 0.005	0.001
Lake age	− 0.003	0.004	− 0.699	0.501	− 0.179	− 0.200	0.023	− 0.002	0.021

A higher value of common effects compared to unique effect also suggests a greater collinearity among explanatory variables. Additionally, negative values can occur in the common effects if some of the relationships among environmental variables have opposite trends. Beta coefficients indicate an environmental variable’s total contribution to the regression equation, whereas structure coefficients are bivariate correlations between a predictor variable and the dependent variable’s score resulting from the regression model. Note that structure coefficients are independent of collinearity among predictor variables (Ray-Mukherjee et al. 2014)

*SE* standard error, *β* beta coefficients, *SC* structure coefficients, *Unique* unique effect of variation for each environmental variable in the regression models, *Common* shared effect of variation for each environmental variable in the regression models, total combined effect (i.e., sum of unique and common effects) of variation for each environmental variable in the regression models

*p* < 0.05: **, higher Common than Unique values (indicating collinearity) in italic font, highest Unique values in each group in bold font, and highest total values in each group are underlined

Considering the climate fraction, the unique effect of elevation range was 15.0%, although this value was not significant (*p* = 0.148). The structure coefficients of elevation range indicated a positive response to the pure climate fraction. Other predictors showed much smaller unique effects on the pure climate fraction. Latitude was the second most important predictor of pure climate fraction, but it also showed considerable level of collinearity with other predictors (i.e., high common effect). The pure spatial location fraction was significantly influenced by elevation range, which contributed 63.0% of the variation. The association between the pure spatial location fraction and elevation range was positive. Other predictors showed a minimal unique effect and/or a large common effect. For the full model, elevation range was the only significant predictor (46.2%), having a positive relationship. Lake age also had a small negative unique effect on the full model.

## Discussion

Single study regions inherently have region-specific environmental gradients (i.e., context dependency) which limits our abilities to draw comprehensive conclusions regarding how these gradients structure local communities across multiple regions and globally (Kraft et al. 2011; Heino et al. 2015a). To overcome this problem, we studied community–environment relationships of lake macrophytes at two metacommunity scales (i.e., within region and across regions) using data sets from 16 regions on six continents. Our study revealed that niche processes related to local lake-level environmental conditions are the dominant force structuring macrophytes within metacommunities. However, our findings also suggest that spatial location, possibly referring to dispersal limitation, is important based on the findings of the across-metacommunities analysis, because species may not be able disperse freely across lakes (Heino et al. 2017a). In addition, elevation range being the only significant predictor influencing the strength of the community–environment relationships across metacommunities suggests that increasing climate variation along with wider elevation range strongly drives the variation in macrophyte communities.

### Environmental filtering prevails, but context dependency occurs within metacommunities

The overall explained variation remained relatively modest in all regions. This has been found in numerous freshwater metacommunities comprising different biological groups (Beisner et al. 2006; O’Hare et al. 2012; Alahuhta and Heino 2013; Heino et al. 2015a). However, we were able to detect subtle patterns in macrophyte metacommunities that existed in most of the study regions. In general, we found that environmental filtering overrode the effects of spatial factors in explaining local communities, but our results conflict with those of other studies conducted in lake ecosystems (Padial et al. 2014; Soininen 2014). We discovered that local environmental variables were more important than spatial location in shaping macrophyte communities in most of the 16 study regions. Thus, our findings lend support to the previous studies on aquatic macrophytes conducted at regional extents (Capers et al. 2010; Alahuhta et al. 2013; Viana et al. 2014), showing that environmental filtering is a dominant force structuring macrophyte metacommunities. To our surprise, we found no differences in this pattern between locally more stable and fluctuating lakes. For example, floodplain lakes of Brazil and China were also mainly explained by environmental filtering, a finding that held across boreal lakes of glacial origin.

The observed dominant role of environmental filtering was found to be rather consistent among the study regions despite their variable spatial extents. This contrasts with earlier findings that suggested that the influence of spatial processes had been expected to increase with increasing extent (Leibold et al. 2004; Soininen 2014; Heino et al. 2015b). The spatial extent of our study regions varied from 260 km^2^ in Norway to 138,000 km^2^ in Sweden, but no systematic increase in the effects of spatial processes was noted along with increasing extent. This outcome may be because environmental gradients often become wider with increasing spatial extent, offering more dimensions for environmental filtering to predominate as long as dispersal remains adequate (Leibold et al. 2004; Heino et al. 2017b).

Spatial processes were most important only in the study regions with highly variable elevation (Morocco and Spain), indicating potential dispersal limitation among the studied lakes within these metacommunities. Mountainous environments may create dispersal obstacles or hinder movement in these two study regions. Similar patterns have been observed for different freshwater organism groups in other topographically diverse regions (Hoeinghaus et al. 2007; Wang et al. 2011). This finding suggests that aquatic macrophyte metacommunities are driven by environmental filtering among lakes when no major dispersal barrier related to topography exists in a region, whereas dispersal limitation is of greater importance in topographically variable regions.

In addition to environmental filtering, lake macrophytes in few regions were affected by climatic forcing, suggesting that other biogeographic effects also contribute to local communities. Although pure climate variables were the most important drivers of macrophyte metacommunities only in coastal lakes of Brazil and New Zealand, the joint effect of climate and spatial location dominated over other fractions in four regions. Climate shows clear geographical trends in relation with latitude, longitude, and elevation at broad extents (Willis and Whittaker 2002), leading to spatial structuring of climate variables as in our study. Temperature affects physiology of aquatic macrophytes by determining, for example, their seed germination as well as onset and rate of seasonal growth (Lacoul and Freedman 2006). Macrophytes are also sensitive to cold temperatures and seasonal variations of temperature (Rooney and Kalff 2000; Netten et al. 2011). In addition, climate may indirectly indicate human colonization (e.g., introduction of alien invasive species and land use) when the colonization has a strong latitudinal or longitudinal gradient (Sass et al. 2010; Alahuhta et al. 2017b). In our study, this kind of phenomenon is possible especially in New Zealand.

These findings within metacommunities may have been influenced to some extent by the limited number of explanatory variables. Additional water chemistry and hydromorphology variables could have increased the importance of local environmental variables at least in some macrophyte metacommunities. For example, alkalinity and maximum colonization depth strongly drive macrophyte community variation in many regions (Lacoul and Freedman 2006; Alahuhta and Heino 2013; Søndergaard et al. 2013); however, these local environmental variables were not available for all the study lakes. Moreover, the water chemistry variables we used are often correlated with many of the local variables absent from our study (Johnson et al. 1997; Wagner et al. 2011). In addition, the use of water instead of atmospheric temperatures might have strengthened the species–climate relationships, although the atmospheric temperatures closely mirror water temperatures in most lakes, especially in unstratified ones (O’Reilly et al. 2015). Despite these possible shortcomings, the environmental variables we utilized were carefully selected to indicate specific ecological responses by lake macrophytes (see Austin (2002) for the ecological rational for variable selection).

### Elevation range explains the strength of the community–environment relationships across metacommunities

We expected that elevation range would strongly affect the strength of the community–environment relationships in the across-metacommunities approach. We found clear support for this hypothesis, as the elevation range significantly explained variation in the climate and spatial location fractions and in the full RDA models. Alahuhta et al. (2017a) discovered that the beta diversity of macrophytes was best controlled by elevation range, which was also related to environmental heterogeneity. They also suggested that temperature variability was one of the fundamental mechanisms behind the patterns detected. Our finding on the relationship between the climate fraction and elevation range similarly indicated that wider elevation range leads to increasing temperature amplitude that, in turn, affects macrophyte communities. This observation highlights the fact that although climate was not the primary driver of macrophytes within a metacommunity at regional extents, its influence is vital across the metacommunities in affecting the strength of the community–environment relationships. In this respect, our results follow the findings from other ecosystems that climate is an important biogeographical characteristic structuring various biological organism groups at the broadest extents. This is likely due to lack of the previous empirical analyses on the community–climate relationships on lake macrophytes at global extents, providing inadequate information on this biogeographical pattern for these organisms.

In addition to the linkage with climate fraction, elevation range was also significantly related to the spatial location fraction. This finding is likely related to dispersal limitation, because wide elevation ranges increase the likelihood of dispersal barriers in the environment. If a dispersal barrier is found in the environment, then an isolated spatial location of local communities hinders possibilities for a community to receive colonists and propagules (Heino et al. 2017a). This outcome follows the ideas of metacommunity ecology that dispersal limitation should exist at the broadest extents (Soininen 2014; Heino et al. 2015b). Moreover, the potential dispersal limitation in macrophyte metacommunities found in this study is highly interesting considering that many macrophyte species have been recorded in more than one continent, suggesting that dispersal limitation has only marginal effect on lake macrophytes (Santamaría 2002; Chambers et al. 2008). In addition, many macrophytes are invasive species, which could overcome dispersal limitation due to the international trade and human-mediated environmental changes (Meyerson and Mooney 2007; Van Kleunen et al. 2015).

Other predictors had only a minimal contribution to any of the across-metacommunities-related fractions. Convex hull area had some influence on the climate fraction; however, the pattern was negative. As expected, latitude was not very strongly related to macrophytes. Latitude was slightly negatively correlated with the climate fraction, although the value of common effects clearly exceeded that of unique effects, indicating collinearity with other predictors. Besides, latitude and longitude acted as suppressors for the spatial location fraction and the full model that had minimal shared variance with the dependent variable, but still made some contribution to the regression model (Ray-Mukherjee et al. 2014). In addition, we found little association between the local environmental fraction and the predictors, suggesting that these biogeographical factors have no effect on the strength of the community–environment relationships. This finding is logical, as local environmental variables (e.g., water chemistry) do not show any clear spatial trend at broad extents, but they can strongly vary even between adjacent water bodies (e.g., Elser et al. 2007).

To our surprise, lake age had no consistent effect on macrophytes across the metacommunities. However, our simple ranked lake age variable may not be sensitive enough to capture historical effects on macrophyte communities. For example, Alahuhta et al. (2018) found that melting of glacial sheet ca. 10 000 years ago created variable local environmental conditions in the boreal landscape, further affecting present-day community composition of lake macrophytes in Finland. On the other hand, basin identity representing historical effects was an important factor explaining variation in the community structure of different freshwater organism groups in boreal lakes and rivers (Heino et al. 2017b). Moreover, we recognize that the present study is the first attempt to account for the historical effects on macrophyte communities at global extents, and therefore, more research on this topic is clearly needed.

## Concluding remarks

Our comprehensive study using data on lake macrophytes from 16 regions at two metacommunity scales (within and across metacommunities) sheds light on their community–environment relationships, which often display variable results when different regions are compared. We found that environmental filtering typically dominated over spatial processing in explaining lake macrophytes within metacommunities. We also discovered that the use of the single metacommunity scale gives inadequate information on the environmental patterns explaining variation in macrophyte communities. For example, macrophyte communities were typically not dispersal limited within metacommunities, but spatial barriers seemed to have hindered the movements of macrophytes in some regions when the results of the across-metacommunities analysis were incorporated. Similarly, climate effects related to elevation range were the only predictor of the strength of the community–environment relationships across metacommunities, although climatic influence was limited within individual metacommunities. These complementary results from two metacommunity scales emphasize the need to integrate community ecology and biogeography when variations in local communities are studied. Our findings provide a greater understanding of community variation and the underlying factors, which should contribute to more efficient management strategies aiming to limit biodiversity loss in freshwater ecosystems.

## Electronic supplementary material

Below is the link to the electronic supplementary material.

Supplementary material 1 (DOCX 60 kb)

## Data Availability

Many of the data sets are from state or national administration, where they can be obtained by request.
