# Supplementary material for: Global patterns in the metacommunity structuring of lake macrophytes: regional variations and driving factors
Source: Oecologia. 2018 Oct 29;188(4):1167–82. doi: 10.1007/s00442-018-4294-0 (PMC6244864; doi:10.1007/s00442-018-4294-0)
Supplement: Supplementary file 1 — Supplementary material 1 (DOCX 60 kb) [file 442_2018_4294_MOESM1_ESM.docx]

**Global patterns in the metacommunity structuring of lake macrophytes: regional variations and driving factors**

Janne Alahuhta^1,2*^, Marja Lindholm^1^, Claudia P. Bove^3^, Eglantine Chappuis^4^, John Clayton^5^, Mary de Winton^5^, Tõnu Feldmann^6^, Frauke Ecke^7, 8^, Esperança Gacia^4^, Patrick Grillas^9^, Mark V. Hoyer^10^, Lucinda B. Johnson^11^, Agnieszka Kolada^12^, Sarian Kosten^13^, Torben Lauridsen^14^, Balázs A. Lukács^15^, Marit Mjelde^16^, Roger P. Mormul^17^, Laila Rhazi^18^, Mouhssine Rhazi^19^, Laura Sass^20^, Martin Søndergaard^14^, Jun Xu^21^, Jani Heino^22^

***Supplementary Material***

Oecologia

^1^ Geography Research Unit, University of Oulu. P.O. Box 3000, FI‒90014 Oulu, Finland.

^2^ Finnish Environment Institute, Freshwater Centre. P.O. Box 413, FI‒90014 Oulu, Finland.

^3^ Departamento de Botânica, Museu Nacional, Universidade Federal do Rio de Janeiro, Quinta da Boa Vista, 20940‒040, Rio de Janeiro, RJ, Brazil.

^4^ Centre d’Estudis Avançats de Blanes (CEAB), Consejo Superior de Investigaciones Científicas (CSIC), C/accés a la Cala St. Francesc 14, 17300 Blanes, Spain.

^5^ National Institute of Water and Atmospheric Research Limited, P.O. Box 11115, Hamilton, New Zealand.

^6^ Centre for Limnology, Institute of Agricultural and Environmental Sciences, Estonian University of Life Sciences, 61117 Rannu, Tartumaa, Estonia.

^7^ Department of Aquatic Sciences and Assessment, Swedish University of Agricultural Sciences (SLU), P.O. Box 7050, SE‒750 07 Uppsala, Sweden.

^8^ Department of Wildlife, Fish and Environmental Studies, Swedish University of Agricultural Sciences (SLU), SE‒901 83 Umeå, Sweden.

^9^ Tour du Valat, Research Institute for the conservation of Mediterranean wetlands, Le Sambuc, 13200 Arles, France

^10^ Fisheries and Aquatic Sciences, School of Forest Resources and Conservation, Institute of Food and Agricultural Services, University of Florida. 7922 NW 71st Street, Gainesville, Florida, 32609, USA.

^11^ Natural Resources Research Institute, University of Minnesota Duluth, 5013 Miller Trunk Highway, Duluth, MN 55811, USA

^12^ Department of Freshwater Protection, Institute of Environmental Protection‒National Research Institute, Krucza 5/11D, 00-548 Warsaw, Poland.

^13^ Department of Aquatic Ecology and Environmental Biology, Institute for Water and Wetland Research, Radboud University, Heyendaalseweg 135, 6525AJ, Nijmegen, The Netherlands

^14^ Department of Bioscience, Aarhus University. Vejsøvej 25, 8600 Silkeborg, Denmark.

^15^ Department of Tisza River Research, MTA Centre for Ecological Research, Bem tér 18/C, H‒4026, Debrecen, Hungary.

^16^ Norwegian Institute for Water Research (NIVA), Gaustadalléen 21, 0349 Oslo, Norway.

^17^  Department of Biology, Research Group in Limnology, Ichthyology and Aquaculture – Nupélia, State University of Maringá, Av. Colombo 5790, Bloco H90, CEP‒87020‒900, Mringá‒PR, Brazil.

^18^ Laboratory of Botany, Mycology and Environment, Faculty of Sciences, Mohammed V University in Rabat, 4 avenue Ibn Battouta B.P. 1014 RP, Rabat, Morocco.

^19^ Moulay Ismail University, Faculty of Science and Technology, Department of Biology, PB 509, Boutalamine, Errachidia, Morocco.

^20^ Illinois Natural History Survey, Prairie Research Institute, University of Illinois, 1816 South Oak Street, Champaign, IL 61820, USA.

^21^ Institute of Hydrobiology, Chinese Academy of Sciences, Wuhan 430070, China

^22^ Finnish Environment Institute, Biodiversity Centre, P.O. Box 413, FI‒90014 Oulu, Finland.

*Corresponding author: Geography Research Unit, University of Oulu. P.O. Box 3000, FI‒90014 Oulu, Finland. Email: janne.alahuhta@oulu.fi, GSM: +358503662601.

Appendix S1. Description of lakes in addition to macrophyte and water quality surveys.

*Brazil, Paraná River*

The sampled lakes in the Upper Paraná River floodplain are characterized as shallow floodplain lakes, which may be permanently connected with the main river channel or may be isolated with a temporary connection with the main river (during the floods). These lakes vary in pH (from acid to alkaline), water transparency (from clear to turbid water) and nutrient concentration (from oligotrophic to eutrophic). In all lakes, we recorded aquatic macrophytes presence by boat at a low speed along the entire lake shoreline. We also carried out species recordings on foot in the shoreline using a transect. We used a grapple, treble hooks and a rake to record submersed species. In this area, we sampled 29 lakes quarterly during 2010.

Total phosphorus and Secchi depth were mean values of four samples. The individual samples were surveyed during the whole floodplain hydrological cycle (March, June, September, December).

*Brazil, coastal lakes*

The studied lakes were part of the Salga project, where lakes from Argentina, Uruguay and Brazil were sampled. For this study, we selected 28 lakes located most close to each other in Brazil for the analysis. The lakes were situated along the coast of Brazil and were small, shallow (mean depth <4.5m) and varied greatly in the degree in which they were impacted by anthropogenic pressures (i.e., agriculture and urban development) (Kosten et al., 2009a). Lake macrophytes were surveyed based on observations along three to eight parallel transects perpendicular to the maximum length of the lake and an additional 20 randomly located points. The number of transects varied with the shape and size of the lake. Observations were made from a boat using a rake when necessary (Kosten et al., 2009b; Kosten et al., 2009c). The surveys were done during summer (lakes at latitudes below 30^o^S) or during dry season (lakes above 30^o^S) between November 2004 and March 2006 by the same team.

All lakes were sampled once during summer (cold and intermediate lakes) or dry season (warm lakes) between November 2004 and March 2006 by the same team. We collected depth-integrated water samples at 20 random points in each lake. From each point sample, 2 l were used to pool into a single large bulk sample. Subsequent subsampling for TP were then frozen until analysis. Total phosphorus (TP) concentrations were analyzed using a continuous flow analyzer (Skalar Analytical BV) following NNI protocol (1986), with the exception of the UV/persulfate destruction which was not executed beforehand but integrated in the system. Secchi depth measurements were conducted at noon in the center of the lake.

*China*

The Yangtze River floodplain is among the most species-rich environments in China because of their fluvial dynamics creating an intricated mosaic of habitats and gradients of hydrological connectivity. A vast extension (15,770 km2) of the mid and lower Yangtze River floodplain is covered by numerous lakes characterized by their shallow, flat and large basins. The surveys were done over the period from 2008 to 2012. Species occurrence in the lakes was assessed on foot along the lake shore and by boat through transects. For each lake, triplicate macrophyte samples were collected by rake with hooks at 0, 0.5, 1.0 m intervals at each of the10-20 transects established randomly for every 20 km^2^ of lake surface area. Each lake was sampled during the summer and autumn, and was visited at least twice during the studied period (i.e., a minimum of 4 surveys per lake). This procedure was assumed to control for the intra-annual variation in macrophyte occurrence.

Total phosphorus and Secchi depth were mean values from multiple transects taken during macrophyte survey.

*Denmark*

The lakes were part of the larger data set consisting of ca. 50 water bodies. Most of the Danish lakes included are located in central Jutland. Most lakes were mesotrophic to eutrophic, alkaline systems with average depth ranging from 0.5 to 16 m. A few humic and low alkaline (i.e., neutral pH or acid) lakes were included, too. Macrophytes were surveyed in an area dependent number of observations point, ranging from 75 to 375 points, situated on equidistant transects covering the entire lake area (if shallow) or the potential macrophyte covered area (if deeper) (Johansson and Lauridsen, 2014). Observation points were distributed ensuring similar observation numbers in each depth interval (0.25 to 1 m depth intervals). A relative species distribution, the total species list and percentage coverage were generated for each lake. Surveys were performed between July 1st and August 15th at maximum macrophyte biomass and before senescence, during the period between 2001 and 2010.

Total phosphorus and Secchi depth were mean values of multiple samples taken between 2001 and 2010. The individual samples were surveyed during the growing season (May-September).

*Estonia*

The selected lakes are part of a larger database of Estonian small lakes. According to the Estonian lake classification, the selected 28 lakes belong to type II (altogether 8 types) and can be considered as a relatively common lake type. This type is characterized by the following parameters: the water area less than 10 km^2^, the average hardness of water (80-240 HCO_3_ mg / l, conductivity 165-400 μS / cm), chloride poor water (chloride content up to 25 mg / l) and without stratification, regardless of the brightness or darkness of the water. The aquatic plant research methodology is characterized by a survey of the entire lake and the assessment of the various species abundance. Most of the selected lakes are located in the south-eastern and southern parts, and few lakes are located to the north and west of Estonia.

Total phosphorous and Secchi depth were average values of May, July, August and September taken in the same year as macrophyte sampling was executed. The average values were based on one sampling point per month in the deepest part of the lake.

*Finland*

A large data set of 150 lakes across Finland have been gathered and maintained by the Finnish Environment Institute. We randomly selected 29 lakes for the study. The majority of the studied lakes were shallow, small humic lakes, and many of them were impacted by anthropogenic pressures (i.e., agriculture and urban development). Lake macrophytes were surveyed using a main belt transect method (Kanninen et al., 2013), in which a five-metre-wide transect extends (perpendicularly to the shoreline) from the upper eulittoral to the outer depth limit of vegetation. The transect is divided into zones according to the dominant life-form or species. The number of transects varied depending on lake size. Lake macrophytes are observed by wading or by boat, with the aid of rake and hydroscope. The surveys were done between June and September over the period between 2006 and 2012.

Total phosphorus and Secchi depth were mean values of multiple samples taken between 2006 and 2012. The individual samples were surveyed during the growing season (May-September).

*Florida (US state)*

The 29 randomly selected water bodies were part of the state-wide database of lakes situated in Florida. The study lakes ranged from oligotrophic to hypereutrophic with average chlorophylls ranging from < 1.0 µg/L to over 150 µg/L. The lakes were generally shallow and small. Plant sampling was conducted during summer months between 1991 and 2013 with a varying number of transects per lake depending on the size of the lake (generally 10 transects per lake). The transects were placed uniformly around the lake and went from open water through the littoral zone of each transect. Plants were collected with divers in deep-water areas and rakes in shallow areas and all identified to species.

Total phosphorus and Secchi depth values were the annual mean of monthly (January through December) samples collected during the year when aquatic plant data were measured.

*Hungary*

We randomly chose 30 lakes to this study from a lake pool of ca. 50 lakes. All the studied lakes are small, shallow lakes being located in an agricultural landscape. Lake macrophytes were surveyed using a transect method. The number of transects parallel to shoreline varied according to lakes size (Schaumburg et al., 2007). All parallel transects contained minimum of four belt transects (two metres wide and perpendicular to the shoreline) extended from the upper eulittoral to the outer depth limit of vegetation. Field surveys were conducted between June and September over the period 2004-2012 (Lukács et al. 2015).

Total phosphorus and Secchi depth were individual samples surveyed at the time of the macrophyte sampling during the growing season (May-September).

*Minnesota (US state)*

The used 30 study lakes were part of the large database of 1500 lakes surveyed between 1992 and 2003 by the Minnesota Department of Natural Resources (Section of Fisheries). Aquatic macrophyte data from the ecoregion of Laurentian Mixed Forest Province were used in this study. We randomly chose these study lakes among all lakes situated in this ecoregion. The aquatic plant species were sampled between July and August using a transect method, in which transects were evenly placed around the lake. The 6-m wide transects ran perpendicular to the shore to the maximum depth of vegetation cover, and the number of transects varied depending on lake size. The species were identified from a boat with the help of a grapple.

Total phosphorus and Secchi depth values were based on the average value of multiple samples taken in 2004. However, these multiple samples from a single year correlated strongly with the long-term water quality data (Spearman’s *r* > 0.82; Alahuhta 2015).

*Morocco*

Most of the 29 studied lakes were shallow and were located in the mountains (Middle and High Atlas) or Atlantic plains. These lakes are used by local people for cattle grazing, recreation, water supply and medicinal plants. Lake macrophytes were surveyed on zones (3 x 3 meters) distributed along 2 permanent transects at right angles to one another. The number of zones varied between lakes according to their size. The distance between zones was three to five meters. All species (amphibious, aquatic) were inventoried; however, only hydrophytes were used in the analyses. Field surveys were executed between February and July over the period 2005-2013.

Total phosphorus and Secchi depth were mean values of multiple samples taken between 2005 and 2014. The individual samples were surveyed during the growing season (April-July).

*New Zealand*

This data set was part of a large lake data set maintained by the National Institute of Water and Atmospheric Research Limited. For this study, we randomly chose 30 lakes situated in both the North and South Island. Macrophyte data were surveyed using the “Quick Survey Method” of Clayton (1983). Surveyed lakes represented natural water bodies. As a result, the lakes included mostly those of volcanic origin, glacial formation and dune-formed lakes. Lake surveys were biased towards larger waterbodies located in accessible and populated areas. Consequently lakes included those influenced by anthropogenic nutrient enrichment or by the introduction of alien plants and fish. The timing of surveys was primarily in the austral spring to autumn (November to April); however, most submerged species are perennial and present year-round in New Zealand. Between one and 50 sites were assessed per lake, with generally more sites in larger lakes. At each site scuba divers covered a 2 m wide transect from the shoreline water level to the deepest extent of vegetation, identifying all plant species seen, with the exception of bryophytes.

Total phosphorus and Secchi depth were mean values from all available sampling events taken at each lake (n = 6 to 89) within the period 2005-2009.

*Norway*

The studied 29 high-alkalinity lakes are small in surface area, varying from oligotrophic to eutrophic status and subject to agricultural land use pressure. These lakes situate in Nord-Trøndelag county in the middle of Norway. Lake macrophytes were surveyed along four orthogonal transects perpendicular to the lake shore and situated approximately at its intersection with the four cardinal points (Viana et al., 2014). In addition, the rest of the lake and its edges were visited, and any additional species recorded. The surveys were carried out during the peak of the growing season in 1998, so that all species present in the lake through the season could be detected. Only hydrophytes were used in the analyses.

Total phosphorus and Secchi depth values represent one single sample for each lake, sampled in September 1998.

*Poland*

Lakes randomly chosen from a national lake pool located in lowlands (<200 m a.s.l.), with high-alkalinity and clear waters waters, but differ in morphometry and trophy. Data on macrophytes were collected in the period from 2004 to 2012 within the national lake monitoring programme (425 lakes) and other research projects. Lake macrophytes were surveyed between June and September using the unified field survey procedure based on belt transect method (Ciecierska and Kolada, 2014). The number of transects varied depending on lake size; however, sampling effort has not influenced previous studies on macrophyte community compositions (Kolada et al, 2014). Within the phytolittoral zone of each lake, the maximum colonisation depth, the mean vegetation coverage and the relative cover of all the aquatic and emergent plant communities were determined. Only hydrophytes were used in our work.

All macrophyte surveys were accompanied with the local environmental variables (here TP and Secchi depth reading used), which were measured four times within the growing season between April and October in the same year as macrophytes were surveyed.

*Spain*

30 lakes out of 66 were randomly chosen in Spain. Macrophytes were sampled between 2005 and 2009 across Catalonia (NW Spain). The data set includes a diversity of water body typologies from alpine lakes (at high altitude with oligotrophic soft-waters), karstic lakes (high alkaline waters), coastal lagoons (at the shoreline, with brackish waters), permanent ponds and temporary pools (small ponds with annual desiccation period). Water bodies were usually small and shallow and were located along a large altitudinal range (0 to 2573 m a.s.l.). Sampling was conducted at the end of the growing period, from late spring to mid-summer depending on the water mass altitude, by snorkeling, scuba diving or walking with waders around all the water body and collecting macrophyte samples at the different assemblages that were recognized by visual inspection (Chappuis et al., 2014).

The coefficient of light extinction (λ) was measured with a Biospherical QSI-140B quantum scalar PAR meter and further converted to Secchi depth (D_secchi_) following D_secchi_ = 1.7 / λ. However, in many lakes, the Secchi depth was more than lake maximum depth. In this kind of lakes we used maximum depth as the value for Secchi depth.

*Sweden*

The studied 30 lakes varied in their environmental conditions and subjectivity to anthropogenic pressures. The randomly chosen lakes were part of a national database covering the whole country. Macrophyte surveys were conducted between 2008 and 2013 using a transect method, in which the transects were placed perpendicular to the shoreline and distributed around the lake, from the upper eulittoral to the outer limit of vegetation. The number of transects varied according to lake size with larger lakes having more transects and vice versa. Species were identified by diving along the transects with 0.5m in 20-cm depth intervals and in plots of ca. 25 × 50 cm (Naturvårdsverket, 2010).

Total phosphorus and Secchi depth were based on a single water sample executed simultaneously with the macrophyte surveys.

*Wisconsin (US state)*

The dataset of 30 lakes for Wisconsin, USA, was chosen from 53 originally surveyed lakes of glacial origin. The lakes generally followed increasing tropic levels, linearly related to both decreasing latitude and increasing anthropogenic pressures. Lake surface area was limited from 20 to 136 ha and watershed size was limited to 27–2205 ha. Macrophyte communities were sampled at each lake once from 2003 to2005 at 14 random-stratified locations to ensure equal sampling of developed and undeveloped terrestrial shoreline. Bog and wetland areas were avoided. Plants were identified within 0.25 m^2^ squares located in 2 to 3m intervals along transects placed perpendicular to shore (Sass et al. 2010).

Total phosphorus and Secchi depth values were collected at turnover in the same year macrophytes were sampled (Sass et al. 2010). Total phosphorus was taken at a depth of 1 m at the deepest part of the lake during spring/fall turnover, when Secchi depth was also surveyed.

Appendix S2. Adjusted R^2^ and p values of individual explanatory variables in explaining macrophytes in RDA models when other variable belonging to the same group (i.e., local, climate and spatial location variables) are controlled. Significant values (p≤0.05) are marked using italic font.

|  |  |  | Adj. R^2^ | p |
| --- | --- | --- | --- | --- |
| Brazil Parana river floodplain | Local variables | Total phosphorus | 0.014 | 0.158 |
|  |  | Secchi depth | 0.014 | 0.118 |
|  |  | Lake area | 0.012 | 0.177 |
|  | Climate variables | Mean temperature | *0.025* | *0.033* |
|  |  | Temperature range | 0.003 | 0.318 |
|  |  | Precipitation | 0.008 | 0.207 |
|  | Spatial location variables | Latitude | *0.076* | *0.001* |
|  |  | Longitude | *0.057* | *0.002* |
| Brazil coastal lakes | Local variables | Total phosphorus | *0.039* | *0.035* |
|  |  | Secchi depth | 0.029 | 0.070 |
|  |  | Lake area | <0.001 | 0.625 |
|  | Climate variables | Mean temperature | 0.022 | 0.094 |
|  |  | Temperature range | *0.031* | *0.048* |
|  |  | Precipitation | *0.047* | *0.006* |
|  | Spatial location variables | Latitude | <0.001 | 0.780 |
|  |  | Longitude | <0.001 | 0.617 |
| China | Local variables | Total phosphorus | <0.001 | 0.803 |
|  |  | Secchi depth | *0.023* | *0.032* |
|  |  | Lake area | 0.011 | 0.167 |
|  | Climate variables | Mean temperature | <0.001 | 0.778 |
|  |  | Temperature range | <0.001 | 0.491 |
|  |  | Precipitation | 0.007 | 0.250 |
|  | Spatial location variables | Latitude | *0.023* | *0.046* |
|  |  | Longitude | *0.025* | *0.042* |
| Denmark | Local variables | Total phosphorus | <0.001 | 0.921 |
|  |  | Secchi depth | *0.062* | *0.019* |
|  |  | Lake area | *0.036* | *0.001* |
|  | Climate variables | Mean temperature | 0.006 | 0.299 |
|  |  | Temperature range | 0.005 | 0.300 |
|  |  | Precipitation | 0.004 | 0.364 |
|  | Spatial location variables | Latitude | 0.001 | 0.419 |
|  |  | Longitude | 0.005 | 0.285 |
| Estonia | Local variables | Total phosphorus | *0.038* | *0.006* |
|  |  | Secchi depth | *0.032* | *0.010* |
|  |  | Lake area | <0.001 | 0.510 |
|  | Climate variables | Mean temperature | 0.012 | 0.128 |
|  |  | Temperature range | *0.030* | *0.011* |
|  |  | Precipitation | *0.021* | *0.047* |
|  | Spatial location variables | Latitude | *0.032* | *0.007* |
|  |  | Longitude | *0.034* | *0.009* |
| Finland | Local variables | Total phosphorus | 0.004 | 0.341 |
|  |  | Secchi depth | *0.032* | *0.003* |
|  |  | Lake area | 0.017 | 0.059 |
|  | Climate variables | Mean temperature | *0.023* | *0.022* |
|  |  | Temperature range | 0.010 | 0.149 |
|  |  | Precipitation | *0.057* | *0.002* |
|  | Spatial location variables | Latitude | 0.018 | 0.076 |
|  |  | Longitude | *0.022* | *0.044* |
| Florida | Local variables | Total phosphorus | *0.048* | *0.004* |
|  |  | Secchi depth | *0.043* | *0.002* |
|  |  | Lake area | <0.001 | 0.549 |
|  | Climate variables | Mean temperature | <0.001 | 0.756 |
|  |  | Temperature range | <0.001 | 0.757 |
|  |  | Precipitation | <0.001 | 0.583 |
|  | Spatial location variables | Latitude | <0.001 | 0.454 |
|  |  | Longitude | <0.001 | 0.766 |
| Hungary | Local variables | Total phosphorus | 0.006 | 0.301 |
|  |  | Secchi depth | 0.008 | 0.225 |
|  |  | Lake area | <0.001 | 0.495 |
|  | Climate variables | Mean temperature | 0.015 | 0.093 |
|  |  | Temperature range | 0.019 | 0.054 |
|  |  | Precipitation | 0.018 | 0.066 |
|  | Spatial location variables | Latitude | 0.001 | 0.428 |
|  |  | Longitude | 0.003 | 0.361 |
| Minnesota | Local variables | Total phosphorus | *0.028* | *0.025* |
|  |  | Secchi depth | 0.008 | 0.196 |
|  |  | Lake area | 0.019 | 0.069 |
|  | Climate variables | Mean temperature | *0.047* | *0.003* |
|  |  | Temperature range | 0.015 | 0.073 |
|  |  | Precipitation | *0.025* | *0.010* |
|  | Spatial location variables | Latitude | 0.009 | 0.173 |
|  |  | Longitude | *0.034* | *0.008* |
| Morocco | Local variables | Total phosphorus | 0.002 | 0.360 |
|  |  | Secchi depth | <0.001 | 0.986 |
|  |  | Lake area | *0.059* | *0.002* |
|  | Climate variables | Mean temperature | 0.014 | 0.118 |
|  |  | Temperature range | 0.024 | 0.058 |
|  |  | Precipitation | *0.041* | *0.013* |
|  | Spatial location variables | Latitude | *0.096* | *0.001* |
|  |  | Longitude | *0.085* | *0.001* |
| New Zealand | Local variables | Total phosphorus | 0.013 | 0.130 |
|  |  | Secchi depth | 0.006 | 0.258 |
|  |  | Lake area | <0.001 | 0.720 |
|  | Climate variables | Mean temperature | 0.012 | 0.130 |
|  |  | Temperature range | *0.036* | *0.004* |
|  |  | Precipitation | 0.006 | 0.244 |
|  | Spatial location variables | Latitude | *0.035* | *0.010* |
|  |  | Longitude | *0.079* | *0.001* |
| Norway | Local variables | Total phosphorus | *0.041* | *0.013* |
|  |  | Secchi depth | 0.014 | 0.146 |
|  |  | Lake area | <0.001 | 0.775 |
|  | Climate variables | Mean temperature | 0.011 | 0.177 |
|  |  | Temperature range | *0.045* | *0.012* |
|  |  | Precipitation | *0.040* | *0.010* |
|  | Spatial location variables | Latitude | 0.018 | 0.096 |
|  |  | Longitude | 0.024 | 0.068 |
| Poland | Local variables | Total phosphorus | 0.005 | 0.277 |
|  |  | Secchi depth | *0.026* | *0.031* |
|  |  | Lake area | 0.012 | 0.165 |
|  | Climate variables | Mean temperature | <0.001 | 0.400 |
|  |  | Temperature range | *0.029* | *0.016* |
|  |  | Precipitation | *0.040* | *0.007* |
|  | Spatial location variables | Latitude | *0.056* | *0.001* |
|  |  | Longitude | *0.024* | *0.022* |
| Spain | Local variables | Total phosphorus | 0.002 | 0.402 |
|  |  | Secchi depth | 0.009 | 0.241 |
|  |  | Lake area | *0.045* | *0.019* |
|  | Climate variables | Mean temperature | <0.001 | 0.516 |
|  |  | Temperature range | 0.025 | 0.087 |
|  |  | Precipitation | 0.022 | 0.107 |
|  | Spatial location variables | Latitude | *0.057* | *0.011* |
|  |  | Longitude | *0.061* | *0.004* |
| Sweden | Local variables | Total phosphorus | *0.041* | *0.003* |
|  |  | Secchi depth | *0.027* | *0.008* |
|  |  | Lake area | <0.001 | 0.610 |
|  | Climate variables | Mean temperature | 0.013 | 0.116 |
|  |  | Temperature range | 0.014 | 0.093 |
|  |  | Precipitation | *0.023* | *0.044* |
|  | Spatial location variables | Latitude | *0.029* | *0.018* |
|  |  | Longitude | *0.034* | *0.010* |
| Wisconsin | Local variables | Total phosphorus | 0.022 | 0.064 |
|  |  | Secchi depth | <0.001 | 0.926 |
|  |  | Lake area | <0.001 | 0.478 |
|  | Climate variables | Mean temperature | *0.027* | *0.014* |
|  |  | Temperature range | 0.018 | 0.059 |
|  |  | Precipitation | <0.001 | 0.710 |
|  | Spatial location variables | Latitude | *0.059* | *0.002* |
|  |  | Longitude | 0.009 | 0.158 |

Appendix S3. Results of the variation partitioning (results shown as adjusted R^2^ values) based on partial redundancy analysis (pRDA) in explaining the relationship between lake macrophyte communities and three environmental variable groups (i.e., local variables, climate variables and principal coordinates of neighbor matrices analysis (PCNM)) in each study region. Separate pRDA analysis using identical explanatory variables was done for each study region. Local and climate variables were forced to the models, whereas PCNM variables were selected based on Blanchet et al. (2008). Significant (p < 0.05) pure fractions are bolded.

|  | Local variables (LV) | Climate variables (CV) | PCNM | LV+CV | CV+PCNM | LV+PCNM | LV+CV+PCNM | Unexplained variation |
| --- | --- | --- | --- | --- | --- | --- | --- | --- |
| Brazil, Parana river floodplain | 0.001 | -0.012 | -0.017 | 0.025 | 0.069 | 0.013 | 0.005 | 0.917 |
| Brazil, coastal lakes | -0.017 | -0.013 | 0.000 | 0.025 | 0.000 | 0.000 | 0.000 | 1.006 |
| China | **0.105** | -0.020 | 0.016 | -0.008 | 0.044 | 0.002 | -0.014 | 0.875 |
| Denmark | -0.017 | -0.013 | 0.000 | 0.025 | 0.000 | 0.000 | 0.000 | 1.006 |
| Estonia | **0.045** | 0.009 | -0.003 | 0.021 | 0.043 | 0.000 | -0.010 | 0.895 |
| Finland | **0.059** | **0.043** | **0.032** | 0.033 | -0.014 | -0.006 | 0.007 | 0.846 |
| Florida | **0.876** | 0.022 | **0.043** | -0.004 | -0.029 | 0.034 | 0.006 | 0.841 |
| Hungary | 0.026 | 0.031 | 0.029 | 0.007 | 0.053 | 0.012 | -0.018 | 0.860 |
| Minnesota | 0.011 | 0.014 | 0.005 | 0.005 | 0.082 | 0.012 | 0.013 | 0.858 |
| Morocco | -0.003 | **0.053** | **0.176** | 0.005 | 0.062 | 0.028 | 0.035 | 0.643 |
| New Zealand | 0.010 | **0.091** | **0.025** | 0.009 | -0.006 | -0.002 | 0.013 | 0.860 |
| Norway | 0.025 | 0.033 | **0.041** | 0.014 | 0.026 | 0.026 | 0.008 | 0.827 |
| Poland | 0.022 | 0.005 | -0.003 | 0.018 | 0.038 | 0.005 | 0.017 | 0.899 |
| Spain | 0.043 | -0.001 | 0.023 | 0.018 | 0.037 | 0.000 | 0.002 | 0.878 |
| Sweden | **0.064** | 0.001 | -0.005 | 0.025 | 0.040 | 0.010 | -0.003 | 0.868 |
| Wisconsin | 0.005 | 0.023 | 0.001 | 0.010 | 0.112 | 0.009 | -0.010 | 0.850 |

Appendix S4. The number of selected PCNMs (in the order of importance) using the forward selection method by Blanchet et al. (2008) in each study region based on overland distances on geographic coordinates of lake centroids.

| Study region | Selected PCNMs |
| --- | --- |
| Brazil, Parana river floodplain | PCNM1, PCNM2 |
| Brazil, coastal lakes | PCNM1, PCNM4 |
| China | No variables selected |
| Denmark | PCNM1 |
| Estonia | PCNM2 |
| Finland | PCNM5 |
| Florida | PCNM4, PCNM3, PCNM2 |
| Hungary | PCNM1, PCNM3, PCNM2 |
| Minnesota | PCNM1, PCNM4, PCNM2, PCNM3 |
| Morocco | PCNM1 |
| New Zealand | PCNM2, PCNM3 |
| Norway | PCNM2 |
| Poland | No variables selected |
| Spain | PCNM1 |
| Sweden | PCNM1, PCNM4 |
| Wisconsin | PCNM1 |

**References**

Alahuhta, J. (2015) Geographic patterns of lake macrophyte communities and species richness at regional scale. Journal of Vegetation Science, 26, 564-575.

Chappuis, E., Gacia, E. & Ballesteros, E. (2014) Environmental factors explaining the distribution and diversity of vascular aquatic macrophytes in a highly heterogeneous Mediterranean region. Aquatic Botany, 113, 72-82.

Ciecierska, H. & Kolada, A. (2014) ESMI: a macrophyte index for assessing the ecological status of lakes. Environmental Monitoring and Assessment, 186, 5501-5517.

Clayton, J.S. (1983) Sampling aquatic macrophyte communities. In: Biggs BJ, Gifford JS, Smith DG ed. Biological methods for water quality surveys. Water and Soil Miscellaneous Publication 54. Wellington, New Zealand, Ministry of Works and Development

Johansson, L. S. & Lauridsen, T. L. (2014) Planteundersøgelser i søer. Teknisk anvisning S04. Aarhus Universitet, DCE Nationalt Center for Miljø og Energi. Available at: http://bios.au.dk/fileadmin/bioscience/Fagdatacentre/Ferskvand/S04_makrofyter_ver2_20140620.pdf

Kanninen, A., Vallinkoski, V.-M., Leka, J., Marjomäki, T. J., Hellsten, S. & Hämäläinen, H. (2013) A comparison of two methods for surveying aquatic macrophyte communities in boreal lakes: implications for bioassessment. Aquatic Botany, 104, 88-100.

Kolada, A., Ciecierska, H., Ruszczyńska, J. & Dynowski, P. (2014) Sampling techniques and inter-surveyor variability as sources of uncertainty in Polish macrophyte based metric for lake ecological status assessment. Hydrobiologia, 737, 256-279.

Kosten S, Huszar, V.L.M., Mazzeo, N., Scheffer, M., Sternberg, L.D.S.L. & Jeppesen, E. (2009a) Lake and watershed characteristics rather than climate influence nutrient limitation in shallow lakes. Ecological Applications, 19, 1791-1804.

Kosten, S., Kamarainen, A., Jeppesen, E., Van Nes, D., Peeters, E.T.H.M., Mazzeo, N., Hauxwell, J., Hansel-Welch, N., Lauridsen, T.L., Søndergaard, M., Bachmann, R.W., Lacerot, G. & Scheffer, M. (2009b) Climate-related differences in the dominance of submerged macrophytes in shallow lakes. Global Change Biology, 15, 2503-2517.

Kosten, S., Lacerot, G., Jeppesen, E., Da Motta Marques, D., Van Nes, E.H., Mazzeo, N. & Scheffer, M. (2009c) Effects of submerged vegetation on water clarity across climates. Ecosystems, 12, 1117-1129.

Lukács, B.A., Tóthmérész, B., Borics, G., Várbíró, G., Juhász, P., Kiss, B., Müller, Z., G-Tóth, L. & Erős, T. (2015) Macrophyte diversity of lakes in the Pannon Ecoregion (Hungary). Limnologica, 53, 74–83.

Naturvårdsverket (2010) Handledning för miljöövervakning - Undersökningstyp: Makrofyter i sjöar. Available at URL: <https://www.havochvatten.se/download/18.64f5b3211343cffddb280004851/Makrofyter+i+sj%C3%B6ar.pdf>

NNI (1986). Water—photometric determination of the content of dissolved orthophosphate and the total content of phosphorous compounds by continuous flow analysis. Normcommissie 390 147 ‘‘Waterkwaliteit’’, Nederlands Normalisatie-insituut, p 8.

Sass, L., M. Bozek, J. Hauxwell, K. Wagner, S. Knight. (2010) Response of aquatic macrophytes to human land use perturbations in the watersheds of Wisconsin lakes, U.S.A. Aquatic Botany, 93, 1–8.

Schaumberg, J., Schranz, C., Foerster, J., Gutowski, A., Hofmann, G., Meilinger, P., Schneider, S. & Schmedtje, U. (2004) Ecological classification of macrophytes and phytobenthos for rivers in Germany according to the water framework directive. Limnologica, 34, 283-301.

Verburg, P., K. Hamill, M. Unwin & J. Abell, 2011. Lake water quality in New Zealand 2010: status and trends. NIWA client report HAM 2010-107, prepared for the Ministry for the Environment. http://mfe.govt.nz/publications/ser/lake-water-quality-in-nz-2010/index.html

Viana, D.S., Santamaria, L., Schwenk, K., Manca, M., Hobæk, A., Mjelde, M., Preston, C.D., Gornall, R.J., Croft, J.M., King, R.A. Green, A.J. & Figuerola, J. (2014) Environment and biogeography drive aquatic plant and cladoceran species richness across Europe. Freshwater Biology, 59, 2096-2106.
